# Brain glucose uptake during transcranial direct current stimulation measured with functional [^18^F]FDG-PET

**DOI:** 10.1007/s11682-019-00195-4

**Published:** 2019-10-10

**Authors:** Christoph Kraus, Andreas Hahn, Helen Sigurdardottir, Benjamin Spurny, Wolfgang Wadsak, Markus Mitterhauser, Marcus Hacker, Siegfried Kasper, Rupert Lanzenberger

**Affiliations:** 1 Neuroimaging Labs (NIL) - PET & MRI & EEG & Chemical Lab, Department of Psychiatry and Psychotherapy, Medical University of Vienna, Wien, Austria; 2 Department of Biomedical Imaging and Image-guided Therapy, Division of Nuclear Medicine, Medical University of Vienna, Wien, Austria; 3 Center for Biomarker Research in Medicine (CBmed), Graz, Austria; 4 Ludwig Boltzmann Institute Applied Diagnostics, Vienna, Austria

**Keywords:** Transcranial direct current stimulation, tDCS, Dorsolateral prefrontal cortex, [^18^F]FDG, Functional PET, Glucose consumption

## Abstract

Previous evidence indicates that transcranial direct stimulation (tDCS) is a neuromodulatory brain stimulation technique. Easy applicability, low side-effects and negligible costs facilitated its wide–spread application in efforts to modulate brain function, however neuronal mechanisms of tDCS are insufficiently understood. Hence, we investigated the immediate impact of tDCS on the brain’s glucose consumption in a continuous infusion protocol with the radioligand 2-[^18^F]fluoro-2-deoxy-D-glucose ([^18^F]FDG) and positron emission tomography (PET). This novel functional PET (fPET) method is capable to reliably detect area-specific and dynamic absolute glucose demand related to neuronal activity in a single molecular imaging session. Fifteen healthy subjects underwent tDCS at 0.5, 1 and 2 mA (mA) at the bilateral dorsolateral prefrontal cortex (dlPFC, cathodal right) for 10 min during functional [^18^F]FDG-PET lasting 70 min. Active stimulation compared to sham did not yield significant changes in glucose consumption at any tested stimulation intensity in this paradigm. Exploratory investigation of aftereffects provided hints for increased glucose consumption with a delay of 5 min at 1 mA in the right posterior temporal cortex. This is the first study investigating changes of glucose consumption in the brain during tDCS. The lack of immediately increased glucose consumption indicates that energy demanding processes in the brain such as glutamatergic signaling might not be immediately increased by tDCS. However, our results implicate the need of fPET investigations for medium-term and long-term effects.

## Introduction

Transcranial direct current stimulation (tDCS) provides a simple and cost-effective neuromodulatory brain stimulation technique and is widely used in neuropsychological research for inducing changes in cortical excitability. Clinical trials suggest efficacy in fibromyalgia, depression without drug resistance or neurorehabilitation, yet a recent consensus statement did not suggest level A recommendations for any clinical applications (Lefaucheur et al. 2017; Schlaepfer et al. 2010). Moreover, there are several negative studies indicating that effects of tDCS could be weak or prone to a vast amount of heterogeneity (Horvath et al. 2016; Horvath et al. 2015).

The procedure might be suited for repetitive home use, which would constitute an attractive extension of therapeutic agents in clinical psychiatry. Yet for approved clinical usage, improvements of the existing method will be necessary whereby identification of a mechanism of action and its optimal utilization could be helpful. The neurophysiological effects of tDCS are attributed to changes in resting membrane potentials towards depolarization or hyperpolarization, whereby anodal tDCS is thought to increase excitability and cathodal tDCS should mediate decreases. But the definite mechanism of action and – in the case of stimulation for antidepressant treatment over the dorsolateral prefrontal cortex (dlPFC) – the location of the elicited neurophysiological changes remains open. Previous neuroimaging studies on the neurophysiological effects of tDCS detected immediate effects of tDCS on cerebral blood flow or blood-oxygen-level-dependent (BOLD) fMRI signals (Lang et al. 2005; Paquette et al. 2011). Moreover, there are indications for aftereffects shortly after stimulation with these techniques as well as with electroencephalography (EEG). Interestingly, a previous magnetic resonance spectroscopy (MRS) study detected increases of prefrontal *N*-acetylaspartate and striatal glutamate + glutamine during bilateral tDCS over the dlPFC (Hone-Blanchet et al. 2016). The montages used here constitute one of the most common stimulation protocols for antidepressant treatment and are indicative of increases in glutamate signaling during tDCS.

Based on a proof-of-principal study (Villien et al. 2014), we validated measurability of task-induced alterations of cerebral glucose consumption with a continuous infusing paradigm of 2-[^18^F]fluoro-2-deoxy-D-glucose ([^18^F]FDG) (Hahn et al. 2016). With this approach, we showed that simple tasks such as finger tapping or visual stimulation elicit increased glucose demand in the corresponding neuroanatomical locations similar to fMRI (Hahn et al. 2016, 2017). A major advantage of this novel fPET method is that dynamic changes of glucose metabolism are measurable during a single PET-scan at whole brain level. In comparison, the assessment of tasks or interventions with a conventional [^18^F]FDG bolus application requires repeated scans, which is accompanied by high intra-subject variability. In addition, for block-design tasks during simultaneous functional [^18^F]FDG PET and fMRI we demonstrated around 10-fold higher percent signal changes with fPET than fMRI (Rischka et al. 2018). Hence, this novel fPET method is perfectly suited to investigate immediate effects of tDCS on the brain’s glucose metabolism without confounding interference from tDCS-induced current flows in MRI (Antal et al. 2014). Therefore, we tested the impact of tDCS at three different stimulation strengths in a continuous infusion [^18^F]FDG-study. Based on close connections between glutamate and the [^18^F]FDG-signal, a previous positive MRS-result (Hone-Blanchet et al. 2016) and increases of repetitive tDCS treatments on [^18^F]FDG with identical electrode positioning (Yun et al. 2016), we hypothesized to detect increases of glucose consumption during tDCS in the dlPFC and connected brain areas.

## Methods

All study related procedures were approved by the institutional review board of the Medical University of Vienna. The study was registered at clinicaltrials.gov (NCT02999607). Safety of tDCS application during PET-scanning was assured by the respective manufacturers before study start.

### Subjects

Fifteen healthy subjects (7 female, age = 25.7 ± 6.9) were recruited by flyer on boards at the Vienna General Hospital (Table 1). Healthiness was assured by a general medical examination including medical history, ECG, laboratory tests (complete blood count, kidney, liver, thyroid hormones, glucose, CRP) and urinary screening for illicit drugs. Psychiatric history was obtained by a psychiatrist with sufficient clinical experience and the structured clinical interview based on DSM-IV (SCID I + II). We based the sample size on a previous study with the same continuous infusion PET protocol. In this study, a task with 10 min eyes open vs. eyes closed and 10 min finger tapping (fingers I-V continuously) in 15 subjects elicited a large enough effect to demonstrate changes in glucose uptake (Hahn et al. 2016, 2017). A previous protocol with similar tasks produced preliminary findings on [^18^F]FDG in three subjects (Villien et al. 2014).

**Table 1:** Subject characteristics

Subjects				p*
n	15			
Sex (f/m)	8/7			
Age (y)	25.7 ± 6.9			
		PET 1	PET 2	
weight (kg)		73.7 ± 17.7	74.2 ± 17.3	0.26
Plasma glucose (mmol/l)		5.2 ± 1.15	5.53 ± 0.79	0.86
Injected dose (MBq)		236.66 ± 53.87	235.88 ± 54.79	0.83
Injected dose/kg body weight (MBq)		3.22 ± 0.16	3.18 ± 0.1	0.6

Numbers are *N* if not otherwise specified. * variables were tested with 2-sided t-tests

### Electrode placement and transcranial direct current stimulation during PET

Direct current stimulation was performed with a NeuroConn DC Stimulator PLUS (neuroCare Group, Munich, Germany) and 5 × 7 cm rubber electrodes covered by saline soaked sponges. To prevent draining and ensure electrical conductance during 70 min scanning, sponges were covered with a thin layer of water and glycerin based electrode gel (Medesign, Dietramszell, Germany). Electrodes were located over the right (cathode) and left dlPFC (anode) by standardized neuronavigation before each PET-scan. The location was chosen since it is the montage, which is used in antidepressant treatment trials (Brunoni et al. 2017; Padberg et al. 2017) For neuronavigation, the subjects’ head was coregistered with the standard brain of the brainsight software (Rogue Research Inc., Montreal, Canada). The center of the electrode was marked and placed over the dlPFC guided by an infrared system. The appropriate MNI (Montreal Neurological Institute) coordinates were taken from the literature (right/left dlPFC: ±38, 44, 26 (Fox et al. 2012)). The electrodes were fixed with a non-conducting net bandage.

**Fig. 1:**
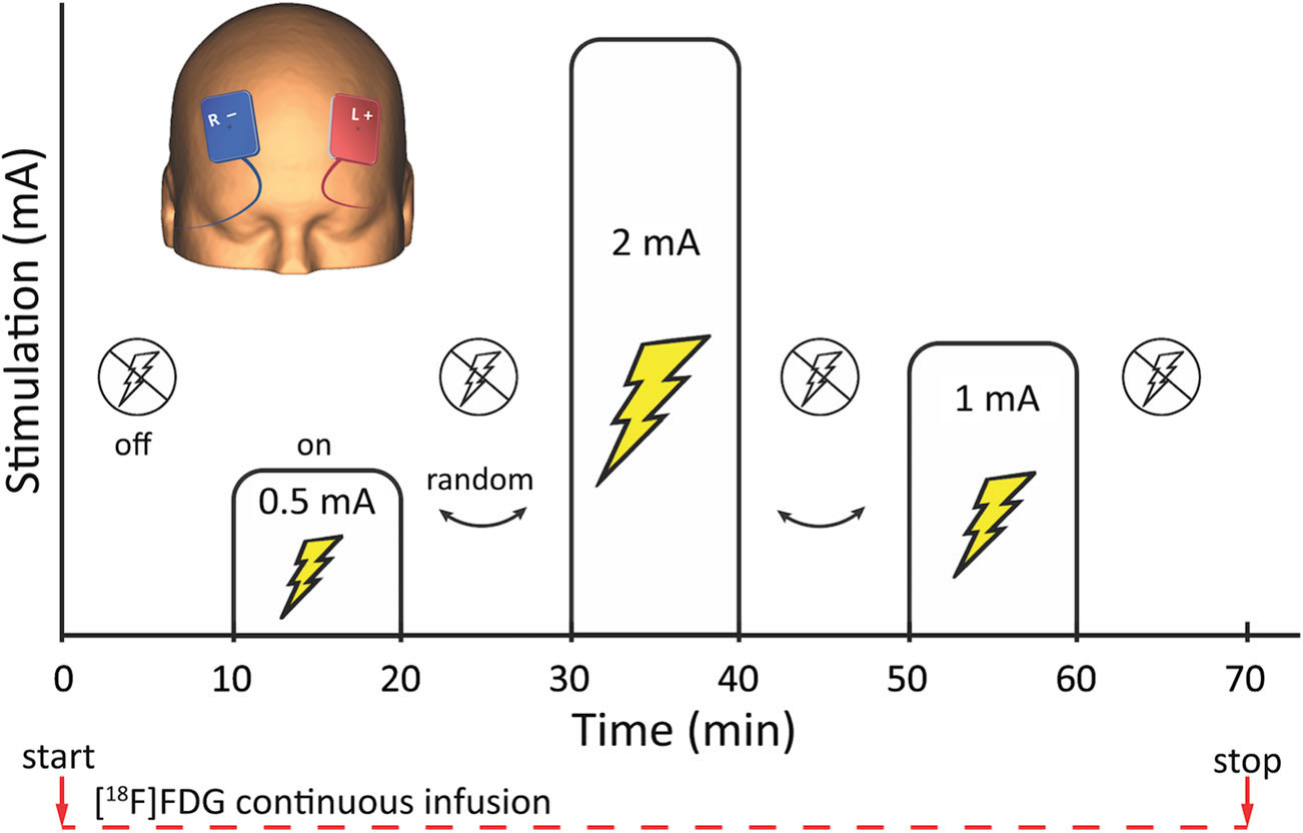
Stimulation setup. A block design analogous to functional MRI was chosen for this functional PET study according to previously published studies with this method. tDCS was performed in a bilateral montage over the dlPFC (anode left) from 10 to 20 min, 30–40 min and 50–60 min at 0.5 mA, 1 mA and 2 mA in between subjects randomized order with 10 min interstimuli intervals. Continuous infusion of [^18^F]FDG at 3 MBq/kg body weight was initiated at the start of scanning for 70 min to measure dynamic changes of glucose consumption at a whole-brain level

Stimulation was performed in a block design during minutes 10–20, 30–40 and 50–60 of PET scanning with 0.5 mA, 1 mA and 2 mA in randomized order (between subjects and scans) during one PET scan (see Fig. 1). A 5 s taper-in and -out period was performed at each beginning and end of stimulation. In a second scan (PET-2) within at least 7 days (average interval: 16.4 ± 10.7 days) sham stimulation was performed. Active and sham was randomized between subjects and scans. Being aware of other methods to conduct placebo tDCS in clinical trials, sham stimulation was conducted by leaving the stimulation in off-mode during the entire scan to ensure no electricity was administered but subjects were told that stimulation was carried out. All subjects were instructed to keep their eyes open and not to move during scanning, which was monitored by a research assistant.

### [^18^F]FDG preparation and imaging procedures

Radiosynthesis of [^18^F]FDG was conducted in-house on each experimental day using a fully automated radiosynthesizer platform (GE FASTlab®, GE Healthcare, USA) with dedicated software and single-use cassettes produced under good manufacturing practice (GMP). [^18^F]FDG preparation followed the well-established nucleophilic substitution route with hydrolysis under basic conditions (Hamacher et al. 1986). Full radiopharmaceutical quality control of [^18^F]FDG according to the respective monograph in the European Pharmacopoeia was conducted before release of the preparation.

All PET scans were performed with a GE Advance full-ring scanner (General Electric Medical Systems, Milwaukee, WI, USA). Subjects were instructed to fast at least 5 h before scanning and only drink water during this period. Patients received i.v. lines for venous blood sampling at one and for tracer application at the opposite arm. Blood glucose was measured from one cubital vein before scanning. Both PET scans started simultaneously with radioligand infusion. PET scans were performed for 70 min with a non-bolus, constant infusion at 3 MBq/kg body weight [^18^F]FDG starting at minute 0 with an infusion rate of 36 mL/h (Hahn et al. 2016, 2017; Villien et al. 2014) with an automated pump (Volumed μVP7000, Arcomed, Regensdorf, Switzerland). Venous blood sampling was performed at minutes 1, 5, 10, 20, 30, 40, 50, 60, and 70. Our previous comparison has yielded that K_i_ estimation with venous glucose levels was comparable to that of arterial ones, so arterial blood sampling could be omitted (Hahn et al. 2016). PET images were reconstructed to consecutive frames of 1 min.

### Image analysis

Preprocessing of whole brain PET images was conducted in SPM12 with default parameters unless specified otherwise. PET images were corrected for head motion (quality = 1) and spatially normalized to the standardized MNI space using a tracer-specific template. PET images were smoothed with an 8 mm Gaussian kernel and a gray matter tissue mask was applied to exclude non-gray matter voxels (SPM12 tissue prior > 0.1). To reduce noise in PET signals (residual scatter, movements) a 12th order FIR low pass filter was applied as a form of temporal smoothing with a cutoff frequency of 5 min (half the duration of the stimulation).

For quantification of tDCS-induced changes of the regional cerebral metabolic rate of glucose (rCMRGlu), a voxel-wise approach in total gray matter was performed following two previous studies from our group. A detailed description of the assumptions and methods of this approach is given there (Hahn et al. 2016, 2017). In short, a general linear model (GLM) was applied to the time activity curves (TACs) of each voxel analogous to functional MRI analyses in Matlab R2011a to separate stimulation effects from baseline uptake. A baseline regressor (*β*_*base*_) was defined as a 3rd order polynomial, while each of the three task regressors (*β*_*task*_) was defined as a linear ramp function with slope = x during differential tDCS strengths (x = 0.5, 1, 2 for 0.5 mA, 1 mA, 2 mA, respectively) and slope = 0 otherwise. Task regressors were orthogonalized to the baseline regressor. Another regressor was included to correct for movement related artifacts (*β*_*move*_). Hereto, a principal component score out of three translation and three rotation parameters was calculated. In a second step, the PATLAK plot was used to estimate K_i_ and rCMRGlu afterwards as described in (Hahn et al. 2016). Finally, percent signal changes (%SC) were computed as %SC = rCMRGlu_stimulation_ / rCMRGlu_baseline_ * 100.

### Statistical analysis

All voxel-wise statistics (in total gray matter) were corrected for multiple comparisons at *p* < 0.05 FWE-corrected cluster level following *p* < 0.001 uncorrected voxel level. Active tDCS induced rCMRGlu %SC in comparison to sham tDCS were tested by repeated measures ANOVA (3 × 2) with stimulation strengths and condition (active/sham) as factors. Post-hoc t-tests compared separate stimulation strengths vs. sham. Upon negative results of the primary outcome, for an explorative analysis of post-stimulation effects, regressors were shifted in time for 3 min and 5 min after start of the stimulation (e.g., min 13, 33, 53 and 15, 35, 55). A more lenient statistical threshold was accepted for explorative analyses (e.g., FDR-correction, or p < 0.001, uncorrected).,

Finally, to investigate potential spatially restricted effects, we conducted a region of interest (ROI) analysis with rCMGlu values extracted from underneath the stimulation sites at the bilateral dlPFC (source: *https://findlab.stanford.edu/functional_ROIs.html*). We repeated identical statistics within the ROIs as in the whole brain analysis.

## Results

There was no significant interaction of treatment (active and sham) × intensity on rCMRGlu %SC (all F-tests *p* > 0.001, uncorrected). Moreover, there was no significant main effect of stimulation vs. sham (*p* > 0.001). Explorative post-hoc t-tests did not yield a significant result between any stimulation strengths separately vs. sham (i.e. 0.5 mA, 1 mA and 2 mA each vs. sham).

**Fig. 2:**
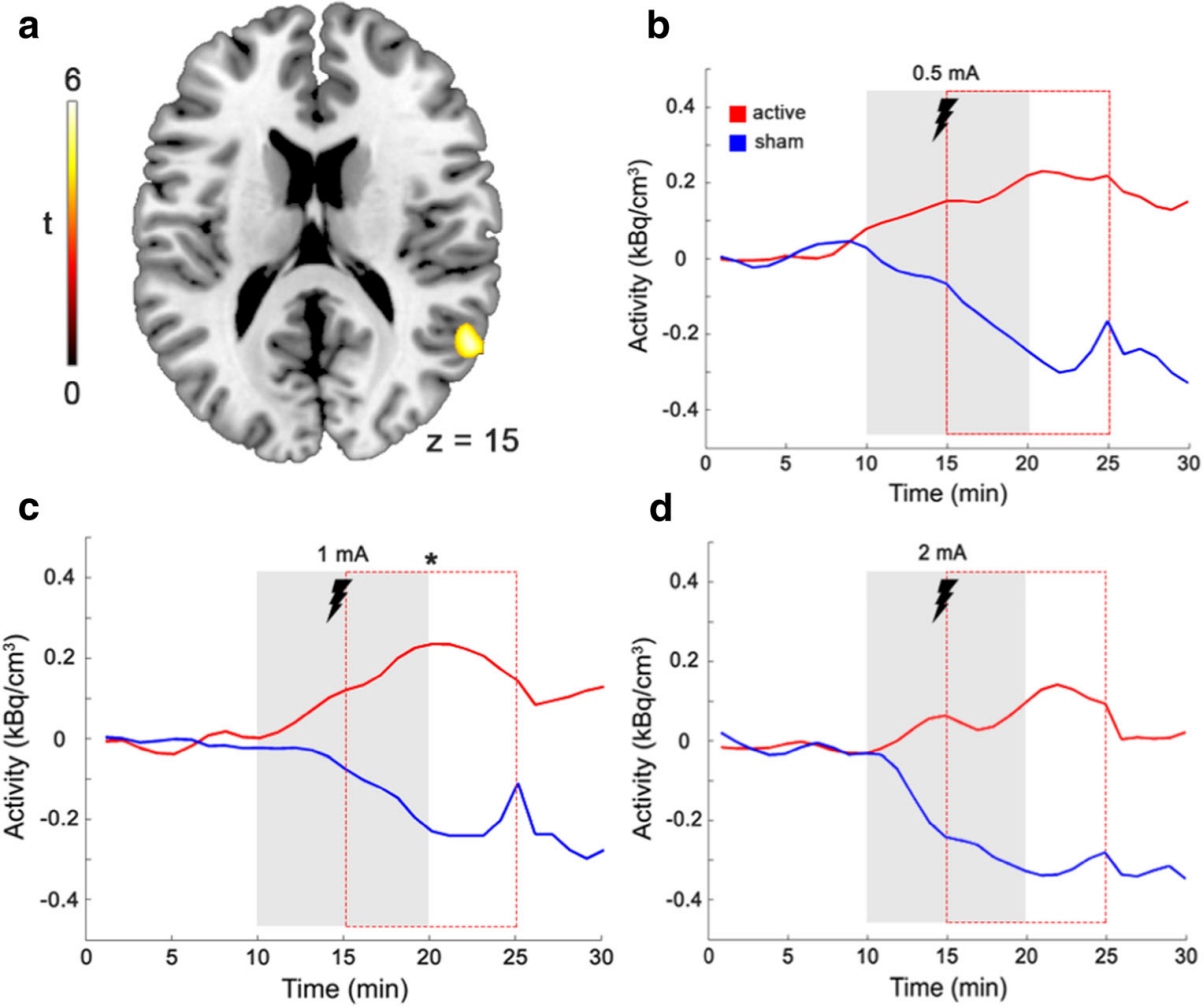
Glucose consumption during tDCS compared sham tDCS. Red line = active tDCS, blue = sham tDCS during stimulation (gray field) and 5 min shifted regressors (red dashed field). Time activity curves were extracted from 70 min total scanning time for each stimulation strength with the respective 10 min pre and post interstimuli intervals (x-axis) and set to zero at time-point 0. Importantly, during stimulation, active tDCS did not elicit significantly different changes in glucose metabolism than sham. In addition, a ROI-analysis (bilateral dlPFC) also yielded negative results (*p* > 0.05). **a** In a post-hoc explanatory analysis we detected trends for increased glucose uptake 5 min after stimulation at 1 mA in the right posterior temporal cortex (t-tests, *p* < 0.05, FDR corrected). z = transversal MNI coordinate. **b**, **c**, **d** Time activity curves of the only significant cluster in the temporal cortex plotted for each stimulation strength. * indicates significance with 5 min time shifts (red dotted line) at 1 mA while 0.5 mA and 2 mA were not significant in post-hoc t-tests

We then tested for post-stimulations effects of tDCS on rCMRGlu %SC by shifting the stimulation model term by 3 min and 5 min. Again, we did not find a significant interaction between treatment × intensity (all F-tests p > 0.001, uncorrected). However, there was a trend for a main effect of stimulus vs. sham for 5 min delay right medial temporal cortex (t = 4.57, cluster size = 223, *p* = 0.098, FDR-corrected cluster level). Exploratory post-hoc t-tests yielded a significant difference of rCMRGlu %SC at 1 mA stimulation (t = 6.18, *p* = 0.03, FDR-corrected cluster level) in the right medial temporal cortex (MNI x, y, z = 56, −54, 20, cluster size = 116, see Fig. 2c). Other stimulation strengths (0.5 mA and 2 mA) at minute 5 did not yield significant results in t-tests (all *p* > 0.001, uncorrected; see Fig. 2b, c). No significant result was obtained for all 3 stimulation strengths at 3 min delay (all *p* > 0.001, uncorrected).

For all 3 stimulation strengths as well as for 3 and 5 min delayed regressors, the ROI analysis underneath the stimulation site did not yield any significant result (*p* > 0.05, uncorrected).

## Discussion

In this functional PET study with a continuous [^18^F]FDG infusion protocol we did not find significant effects of tDCS on simultaneously measured brain glucose metabolism. None of the applied stimulation strengths (0.5 mA, 1 mA and 2 mA) over the dlPFC was associated with changes in cerebral metabolic rate of glucose, neither in whole brain analysis nor in a ROI approach underneath the stimulation sites. Explorative analyses hints towards possible aftereffects. We found significant differences of rCMRGlu with 5 min delay at 1 mA in the right posterior middle and superior temporal cortices. These results suggest that there might be no immediate effects of bilateral dlPFC tDCS on brain glucose uptake, irrespective of stimulation intensity we applied. Rather, our data suggest that there might be aftereffects several minutes after stimulation, which will have to be investigated in further studies.

The method of continuous [^18^F]FDG infusion has the advantage of lacking interference from tDCS-induced current flows in MRI (Antal et al. 2014). With the same technique, we detected robustly increased glucose metabolism after tasks that increase synaptic activity such as finger tapping vs. rest and eyes open vs. eyes closed (Hahn et al. 2016, 2017). Several factors might contribute to these observations. The effect size of tDCS on rCMRGlu changes might be smaller than glucose uptake of finger tapping and vision (Hahn et al. 2016, 2017). A recent study with similar subject number and design on online effects of bilateral tDCS over the dlPFC found immediate increases of prefrontal *N*-acetylaspartate and striatal glutamate + glutamine levels with MRS (Hone-Blanchet et al. 2016). Given that these excitatory effects were driven by increases in glutamate, which are related to glucose demand as measured by [^18^F]FDG-PET (Pfund et al. 2000; Zimmer et al. 2017), we should have detected a similar result. While test-retest ratios between both methods are similar (around 5% (Geramita et al. 2011; Schmidt et al. 1996)), methodological differences between MRS and [^18^F]FDG-PET including measurement with one vs. multiple voxels might account for this contradiction.

The present study is the first to investigate dynamic changes of [^18^F]FDG-PET uptake induced by tDCS in the brain at rest. Functional [^18^F]FDG-PET results yield similar cerebral activation patterns such as task-based fMRI at about 10-fold percent signal changes but lower temporal resolutions (Rischka et al. 2018). The significant advantage compared to bolus infusion PET methods is that only one scan is needed, which reduces test-retest variability and radioligand exposure. Thus, comparisons with PET studies that used other imaging modalities are hard to draw but should be presented here for a complete overview. A previous PET study detailed tDCS’ immediate effects on blood flow (^15^O-water-PET), which were dependent on motor activity in the primary motor cortex (Paquette et al. 2011). Another ^15^O-water study detected aftereffects with more pronounced results at rest compared to movement (Lang et al. 2005). As far as receptor PET is concerned, we are aware of one study investigating acute effects on the μ-opioid receptor selective radioligand [^11^C]carfentanil giving preliminary evidence that the opioid system might be engaged by tDCS but no direct comparison between active and placebo tDCS were reported (DosSantos et al. 2012). A similar study using tDCS showed increased glucose metabolism in the temporal cortex (Yun et al. 2016). Here nine tDCS treatments at 2 mA over a period of three weeks in 16 patients with mild cognitive impairment and identical electrode positions as in our study were conducted. The authors detected increases in [^18^F]FDG-PET uptake in the medial and superior temporal cortices overlapping to our aftereffect result (Yun et al. 2016). The medial and superior temporal cortex were demonstrated to be co-activated with the dlPFC in terms of functional connectivity upon neurostimulation of the dlPFC (Fox et al. 2012). Importantly, our results in healthy subjects do not allow inference on potential effects in patients with prefrontal cortical dysfunction. In sick populations tDCS at the applied stimulation strengths might exhibit altered rCMRGlu reactivity and thus differential results.

Several BOLD-fMRI studies, showed immediate effects as well as aftereffects. Decreases of BOLD responses in the supplementary motor area (SMA) after finger tapping and 20 s tDCS of 1 mA with anodal but not with cathodal tDCS were described (Antal et al. 2011). Further fMRI studies demonstrated effects on resting-state network connectivity in widespread brain areas during bilateral tDCS (SMA1) as well as shortly after stimulation (Keeser et al. 2011; Sehm et al. 2012). Additionally, effects of tDCS with other imaging modalities such as magnetoencephalography (MEG) (Garcia-Cossio et al. 2016), near-infrared spectroscopy (NIRS) and electroencephalography (EEG) (Bergmann et al. 2016) are reported. With EEG and simultaneous tDCS polarity-specific changes of neuronal synchronizations in low frequency bands were demonstrated (Mancini et al. 2016).

Transcranial direct current stimulation is known to exhibit variance attributed to intra- and inter-subject physiological factors such as conductance (Noury et al. 2016), skull permeability, hair or technical factors such as positioning (Guerra et al. 2017). Our “on-off” design over bilateral dlPFC might have introduced variance, too. Aftereffects on neurophysiological parameters such as motor-evoked potentials (MEP) and others were demonstrated after 10–20 min tDCS lasting up to several hours (Jamil et al. 2017). Theoretically, each “on”-block might have affected the following “on”- as well as “off”-blocks. But a positive aftereffect would be detectable in the resting “off”-period between “on”-conditions and might be enhanced by the following “on”-period (i.e. cumulative effects). Indeed, we detected hints towards elevated glucose consumption in the temporal cortex with 5 min delay. However, since our paradigm was not designed for detection of aftereffects, this result calls for further studies systematically probing aftereffects.

The main limiting factor of this study is that the sample size was potentially too small to detect low effect sizes. Nevertheless, the sample size is comparable to previous positive imaging studies with PET and fMRI, but low effect sizes with [^18^F]FDG-PET must be addressed in follow-up studies. An additional limitation is that the “on-off” design might be well suitable for tasks like in our previous functional PET studies but tDCS appears to produce a milieu for facilitated activity after stimulation, so that a single block of stimulation and longer period for aftereffects might be more suitable for future investigations for neuroimaging. Finally, higher stimulation strengths such as 3 mA have yielded stronger effects. Due to the aim to minimize PET scan durations, a 4th stimulation paradigm was not considered during study design and could be investigated in future studies.

In conclusion, with this continuous infusion [^18^F]FDG-PET study we did not detect changes of resting glucose metabolism during 10 min tDCS at 0.5, 1 nor 2 mA. After applying a time shift in an exploratory analysis, there were hints for aftereffects in form of elevated glucose metabolism with 5 min delay in the right posterior temporal cortex. Aftereffects are well established by imaging studies with MRI and PET and electrophysiology. The results of this study warrant further investigations into aftereffects and the influence of tasks and tDCS on glucose consumption.
